# Intellectual disabilitiy in developmental age

**DOI:** 10.1186/1824-7288-41-S2-A38

**Published:** 2015-09-30

**Authors:** Mario Giuffrè, Giovanni Moceri, Davide Vecchio, Vincenzo Antona, Emanuela Salzano, Giovanni Corsello

**Affiliations:** 1 Department of Sciences for Health Promotion and Mother and Child Care “G. D'Alessandro”, University of Palermo, Palermo, 90127, Italy

## 

Intellectual disability (ID) is a neurodevelopmental disorder characterized by deficits in intellectual and adaptive functioning that present before 18 years of age [1]. ID is heterogeneous in etiology and encompasses a broad spectrum of functioning, disability, needs and strengths. Originally formulated in strictly psychometric terms as performance greater than 2.5 SDs below the mean on intelligence testing, the conceptualisation of ID has been extended to include defects in adaptive behaviours [2]. The term-global developmental delay-(GDD) is usually used to describe children younger than 5-years of age who fail to meet expected developmental milestones in multiple areas of intellectual functioning [1]. In both conditions the symptoms must be present in the early developmental period, but they may not become fully manifest until social demands exceed patients’ capacities.

ID affects 1.5 to 2% of the population in Western countries and represents an important health burden [3]. During the past decade, advances in genetic research have enabled genomewide discovery of chromosomal copy-number and single-nucleotide changes in patients with ID and autism as well as in those with other neurodevelopmental disorders. These technological advances-which include array comparative genomic hybridization (CGH), single nucleotide polymorphism genotyping arrays and massively parallel sequencing-have transformed the approach to the identification of etiologic genes and genomic rearrangements in the research laboratory and are now being applied in the clinical diagnostic arena [4]. In this view, the American Academy of Pediatrics recently released a guidance for the clinician in rendering Pediatric Care [5]. The suggested clinical approach to the patient should be conducted closely with a geneticist and includes the child's medical history, the family history, the physical and neurologic examinations (emphasizing the dysmorphology examination) and the examination for neurologic or behavioral signs that might suggest a specific recognizable syndrome or diagnosis. After this clinical evaluation, focused use of genetic laboratory tests, imaging and other consultations are critical in establishing the right diagnosis, its pattern of inheritance and the subsequent follow-up.

Finally, this guidance highlights a renewed emphasis on array CGH, that is now considered the first-line diagnostic test for children who present with GDD/ID of unknown cause, and on the identification of-treatable-causes of GDD/ID with the recommendation to consider screening for inborn errors of metabolism [5]. The future use of whole-genome or next generation sequencing offers promises and challenges needing to be yet addressed before their regular implementation in the clinic.
